# Comparative microleakage outcomes of different techniques used for creating the occlusal anatomy in occlusal direct restorations using the dental operating microscope

**DOI:** 10.1002/cre2.664

**Published:** 2022-10-13

**Authors:** Marius G. Bud, Razvan C. Pop, Razvan Pricope, Anca Mesaros, Andrada Voina, Ada Delean, Ondine Lucaciu, Sanda Cîmpean

**Affiliations:** ^1^ Department of Conservative Odontology, Faculty of Dentistry “Iuliu Hațieganu” University of Medicine and Pharmacy Cluj‐Napoca Cluj‐Napoca Romania; ^2^ Department of Conservative Odontology, Faculty of Dentistry “Iuliu Hațieganu” University of Medicine and Pharmacy Cluj‐Napoca Cluj‐Napoca Romania; ^3^ Department of Prosthodontics and Dental Materials, Faculty of Dentistry “Iuliu Hațieganu” University of Medicine and Pharmacy Cluj‐Napoca Cluj‐Napoca Romania; ^4^ Department of Oral Rehabilitation, Faculty of Dentistry “Iuliu Hațieganu” University of Medicine and Pharmacy Cluj‐Napoca Cluj‐Napoca Romania

**Keywords:** composite brush, dental microscope, direct restoration, layering technique, microleakage, occlusal cavity

## Abstract

**Objectives:**

Microleakage in direct dental restorations is a primary causal factor in the restoration's failure. The aim of this study was to evaluate whether the technique for occlusal layering of the composite resin (the use of brush adaptation, the use of magnification, cusp build‐up, stamp technique) has any effect on microleakage of direct restorations in occlusal cavities.

**Materials and Methods:**

One hundred extracted human molars were restored using five restoration techniques (Packable Bulk technique, Occlusal Stamp technique, Successive Cusp Build‐up technique, Successive Cusp Build‐up technique + Brush adaptation, Successive Cusp Build‐up technique + brush adaptation + Dental Operative Microscope magnification). The teeth were subjected to thermal aging for 800 cycles at 5°C and 55°C, infiltrated with basic fuchsin dye for 24 h, and then sectioned buccolingually in the middle of the crown. Infiltration was measured in four areas of the tooth section by five different observers and then given a score from 1 to 3, proportional to infiltration depth.

**Results:**

The lowest mean scores for infiltration (meaning less infiltration observed) were present in Group A (1.41 ± 0.878) and Group C (1.46 ± 0.679), while Group D showed the highest infiltration scores (1.75 ± 0.853). When comparing the groups for differences, no statistically significant difference in infiltration was found between any technique *p* < .586.

**Conclusion:**

The techniques examined for placing the occlusal layer of composite in direct restorations do not differ significantly in terms of marginal infiltration, although a slight improvement was found when using the bulk technique and the successive cusp build‐up.

## 1 INTRODUCTION

Nowadays, direct composite restorations are one of the most common ways of treating carious lesions on posterior teeth (Cumerlato et al., 2019). Their popularity has increased over time, concurrent with the fall of amalgam restorations (Reher et al., 2021), especially with the advancements made in adhesion and adhesives' long‐term performance, since composite materials alone do not bond directly to the tooth structure (Geerts et al., 2012). Composite resins have also gained more popularity because of their ability to overcome certain drawbacks of older dental restorative materials like amalgam's requirement for a retentive cavity, poor esthetics, and tooth staining (Vanishree et al., 2015). Composite materials, therefore, are able to preserve healthier natural tooth structures by creating a strong chemical bond between enamel and dentin (Zhou et al., 2019).

This type of bonding interface, however, is a weak point of the restoration, since secondary caries is proven to be the main reason for failure in posterior teeth (Demarco et al., 2017). Secondary caries form mostly in cases where a gap between the restoration and the tooth structure is present (Askar et al., 2020). Interface gaps usually form due to the chemical polymerization of the composite's resin, in which monomer molecules come closer to each other, leading to a process called polymerization shrinkage (Shah & Stansbury, 2021). Such gaps, depending on their size, allow acidic fluids along with bacteria to pass between a cavity wall and the restorative material in a process called microleakage (Usha et al., 2011). Microleakage can eventually lead to postoperative sensitivity, marginal staining, or eventual loss of restoration due to secondary caries (Goldstein, 2010). To prevent that, authors suggested different methods for overcoming this problem, one of them focusing on the placement technique of the material (Park et al., 2008).

### 1.1 Restorative techniques described/used in this paper

Bulk technique with simultaneous modeling (Hayashi et al., 2020; Hirata et al., 2015; Veloso et al., 2019) uses a regular consistency low shrinkage bulk fill composite to create the occlusal anatomy by creating the anatomy all at once and then polymerizing the cusps simultaneously. By placing a single increment of composite into the cavity, the necessary time for filling up the cavity is substantially reduced (Scolavino et al., 2016). Although it uses low shrinkage composites, it may cause microleakage by increasing the C‐factor—meaning the ratio of the bonded to unbonded cavity surface areas—and polymerization stress (Ghulman, 2011).

To reduce the C‐factor and relieve the stress created by composite polymerization, the authors describe the successive cusp build‐up technique (Liebenberg, 1996). The occlusal anatomy is reconstructed by placing a small increment of composite, corresponding to each of the tooths' cusps, not touching the opposite wall of the cavity, followed by light curing. By modeling each cusp individually, a good occlusal morphology and a proper marginal adaptation are assured.

The stamp technique (Alshehadat et al., 2016; Perrin et al., 2013) is applied to occlusal caries that do not damage the tooth's occlusal surface. It uses a liquid dam—a flowable and light curable material often used to aid in tooth isolation during a restorative procedure—to take an impression of the tooth's occlusal surface before preparation. The “stamp” is then used for pressing the last layer of composite into shape, stamping the occlusal anatomy. The created anatomy is then polymerized through the impression. By creating an adequate occlusal morphology, similar to the original one, the method offers an accurate functional occlusion, without the need for corrections, with the cost of increasing the C‐factor and polymerization stress and potentially causing microleakage.

The use of a specially designed brush for smoothing and condensing the last layer of the composite may provide a better adaptation to the margins of the cavity, thus decreasing the possibility of marginal infiltration. Nevertheless, more literature support is needed to confirm this hypothesis (Layering composites just got easier, 2018) given the lack of studies supporting the use of a modeling brush for composites, that may decrease microleakage.

The use of a dental operating microscope (DOM) provides better posture and concentration for clinical work and is successfully used for direct restorations (Bud, Jitaru, et al., 2021; Bud, Pricope, et al., 2021). The outcome success of proximal composite restorations may be increased regarding marginal integrity and procedural errors decrease significantly when working with magnification (Leknius & Geissberger, 1995; Reddy et al., 2017). However, there is not enough literature support on this topic yet.

Although the extensive search was conducted through peer‐reviewed literature, no study was found that evaluated whether the technique used for applying the last layer of composite influences the microleakage.

The aim of this study was to evaluate whether the technique for occlusal layering of the composite (the use of brush adaptation, the use of magnification, cusp build‐up) has any effect on the microleakage of direct restorations in occlusal cavities.

## 2 MATERIALS AND METHODS

### 2.1 Selection and tooth preparation

**Figure 1:**
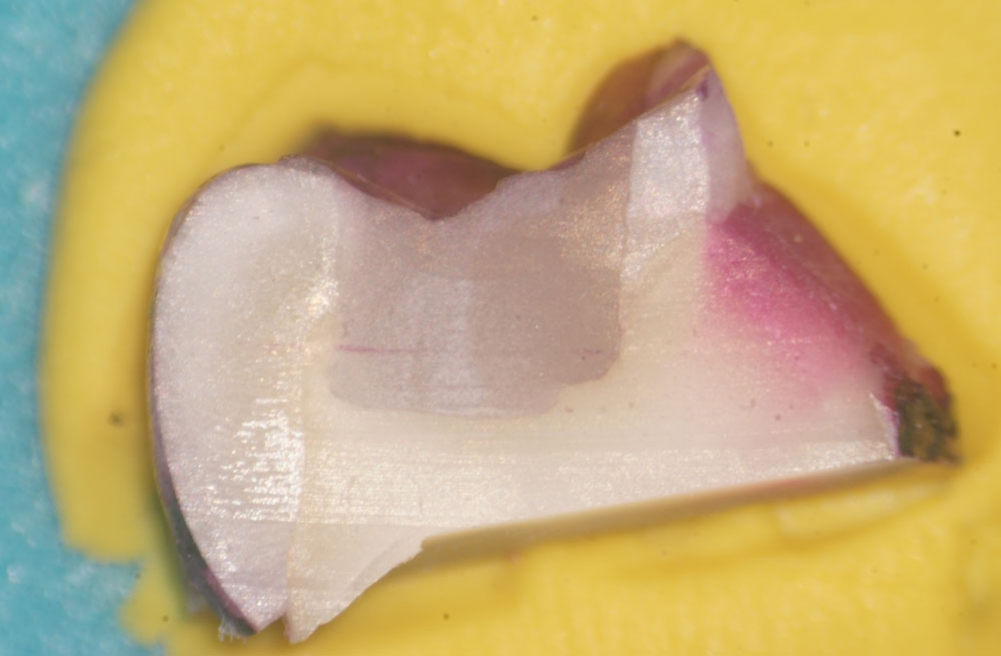
Showing an example of “score 0” infiltration

A sample of 100, caries‐free human extracted molars was used. The teeth were of comparable occlusal sizes of 11.13 mm (±0.72) from mesial to distal marginal crest and 9.1 mm (±0.85) from facial cusp tip to oral cusp tip. Before preparation, the teeth were stored in 0.9% saline solution for 1 month. Standardized cavities were prepared in each tooth on the occlusal surface, with a depth of 3 mm, extended mesiodistally until 1 mm of marginal crest remained intact and buccolingually to ½ of cusp height (Figure 1). Cavities were prepared by means of diamond burs (881‐016‐2X; SS White) mounted to a high‐speed handpiece (NSK), under copious water cooling. The prepared cavities followed the outline of the pits and fissures of the occlusal surface. The teeth were immediately filled with the appropriate technique so as to maximize adhesion to dentin.

### 2.2 Restorative procedure

The 100 teeth were randomly divided into 5 equal groups of 20 samples and assigned to a different composite layering technique, as presented in Table 1. Table 2 presents all composite materials used in this study and their components.

**Table 1:** Showing each layering technique assigned to each group

Group	Layering technique
A	Packable Tetric EvoCeram Bulk Technique + simultaneous technique
B	Flowable bulk (SDR) + packable CeramX + stamp technique
C	Flowable bulk (SDR) + packable CeramX + successive cusp build‐up with no brush adaptation
D	Flowable bulk (SDR) + packable CeramX + successive cusp build‐up with brush adaptation
E	Flowable bulk (SDR) + packable CeramX + successive cusp build‐up with brush adaptation under DOM magnification

Abbreviation: SDR, smart dentin replacement.

**Table 2:** Showing additional information about materials used in the experiment

Composite resin	Type	Producer	Composition	No. of restorations	Batch no.
Tetric EvoCeram Bulk Fill	Packable	Ivoclar Vivadent	Monomer matrix with dimethacrylates (20%–21% weight). Fillers contain barium glass, ytterbium trifluoride, mixed oxide, and prepolymer	20	Z016W4
SDR	Flowable	Dentsply Sirona	Dimethacrylate (EBPADMA), TEGDMA, camphorquinone, hydroxytoluene, UV stabilizer	20	2105000733
Ceram.X SphereTEC one	Packable	Dentsply Sirona	Polysiloxane matrix, poly‐urethane‐methacrylate, bis‐EMA, TEGDMA, SphereTEC fillers, ytterbium fluoride	20	1905000042

Abbreviation: SDR, smart dentin replacement.

Before preparation, each tooth specimen was mounted in a silicon index for stability. There were two individual operators who performed the experiment. One operator was in charge of preparing the standardized cavities and the other was assigned to the restorative procedure. All teeth were restored on a working table, mounted in the silicon index, under conventional LED lighting of a dental unit light (LEDlight Plus, Dentsply Sirona). Only Group E was restored by looking through the DOM with its own lighting.

The etching and bonding protocol was the same for all groups. Etching was performed with 37% phosphoric acid etching gel (Cerkamed) for 30 s on the enamel and 15 s on dentine, followed by abundant washing and gentle drying for 10 s. One layer of the bonding material (Prime Bond Universal, Dentsply Sirona) was applied in the cavity, dried for 5 s with a gentle air blow, and then polymerized for 20 s after the manufacturer's indications, with a LED polymerization lamp (Woodpecker).

For Group A, after condensing the Tetric EvoCeram (lot Z016W4; Ivoclar Vivadent) a packable composite material, the occlusal anatomy was carved with a sharp instrument (LM‐Dental) and then polymerized, using a LED light lamp (Woodpecker) as the manufacturer indicated.

In Group B, cavities were filled to 1.5 mm of the cavity with smart dentin replacement (SDR) bulk flow composite (batch no. 2105000733; Dentsply Sirona) then Ceram.X (lot. 1905000042; Dentsply Sirona) composite was applied in one increment and pressed into shape with the already taken stamp (lot. 2809201; Cerkamed). The restoration was polymerized for 5 s through the stamp and the thread seal tape (Remer Rubinetterie S.p.A) and then for 20 s after the stamp removal.

Groups C, D, and E filled the cavity to 1.5 mm with SDR bulk flow composite, then layered the occlusal anatomy in small increments corresponding to each cusp. In Group C, the composite brush for increment adaptation was not used, in Group D, it was used while working without any magnification and in Group E, the brush was used (001534; GC Corporation) under DOM magnification (Zumax) of ×1.6. Each increment was polymerized for 5 s after layering, with a final polymerization of 20 s. Polymerization was carried out by positioning the tip of the lamp as close to the occlusal surface as possible without touching it.

Each group followed the same finishing and polishing protocol. Finishing was done by means of red olive‐shaped diamond burs (code 368‐020F; SS White) mounted to a high‐speed handpiece (NSK), under water cooling. Polishing was carried out with the use of rubber pieces (code 0304; Kenda) at low speed, followed by polishing brushes (code Y‐019; Kenda) with paste (SuperPolish, Kerr).

After the restoration process was finished, teeth were immersed in 0.9% saline solution for storage.

### 2.3 Tooth aging, infiltration, and measuring

Following the restoration procedure, all teeth were subjected to thermal aging in a thermocycling machine (LTC Group) for 800 cycles in two baths of 5 and 55 C, with a dwelling time of 30 s. After thermal cycling, the teeth' apices were sealed with utility wax and all the teeth surfaces were coated with two layers of nail polish (Farmec), except for 1 mm from the cavity margins. After drying, the teeth were submerged in 0.5% basic fuchsin for 24 h, unexposed to light, and then thoroughly rinsed under running water.

Sectioning was carried out with the use of a sectioning machine (IsoMet Low Speed; Buehler) using a diamond disc at low speed. All specimens were sectioned two times buccolingually in the middle of the tooth, resulting in one tooth cross‐section 2 mm wide. Each tooth cross section's infiltration was evaluated at its four walls mesio‐facially, mesio‐lingually, disto‐facially, disto‐lingually. The scoring is based on a 4 points scale, already proved efficient in other studies (Geerts et al., 2012; Orłowski et al., 2015). It is presented in Table 3.

**Table 3:** The scoring method used for evaluation

Score	Criteria
0	No infiltration
1	Infiltration of the enamel, no more than 1/3 of the cavity depth
2	Infiltration more than 1/3 depth of cavity but no more than 2/3, either dentin or enamel infiltration
3	Dentin infiltration until full depth of cavity

**Figure 2:**
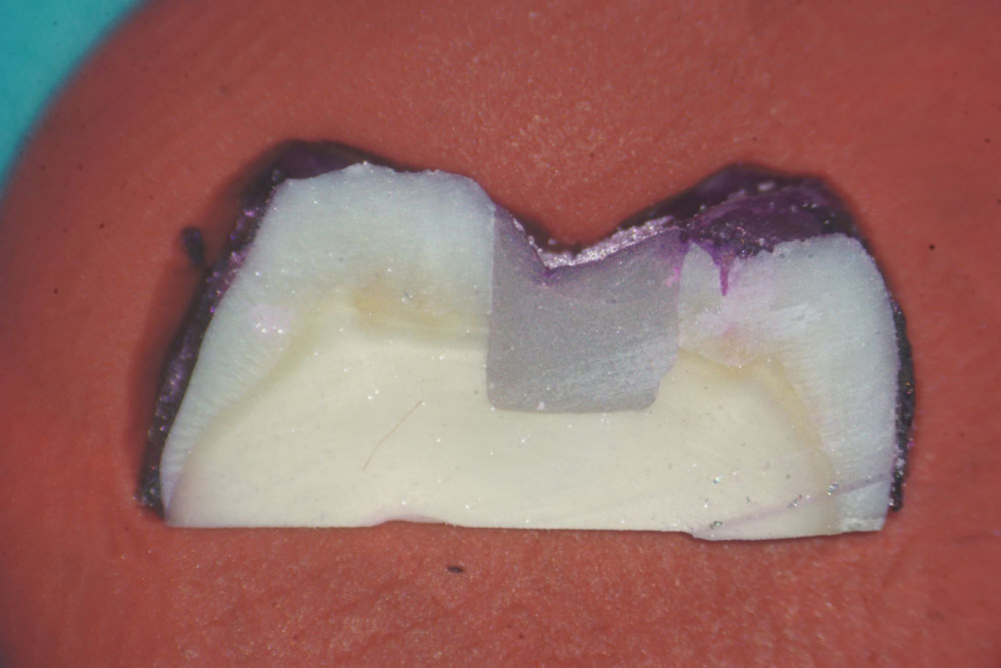
Showing an example of “score 1” infiltration

**Figure 3:**
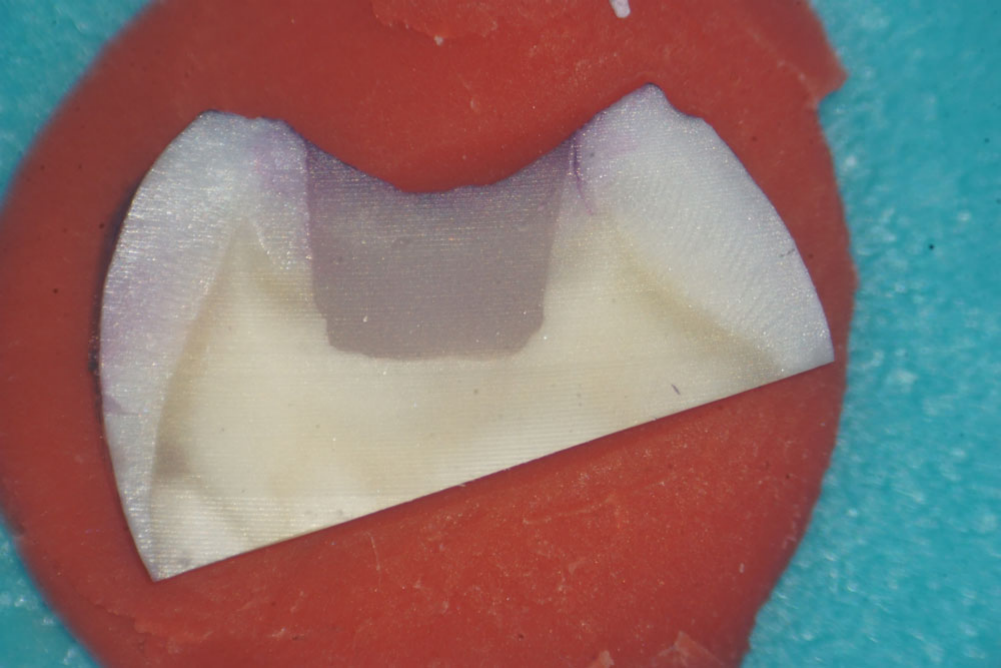
Showing an example of “score 2” infiltration

**Figure 4:**
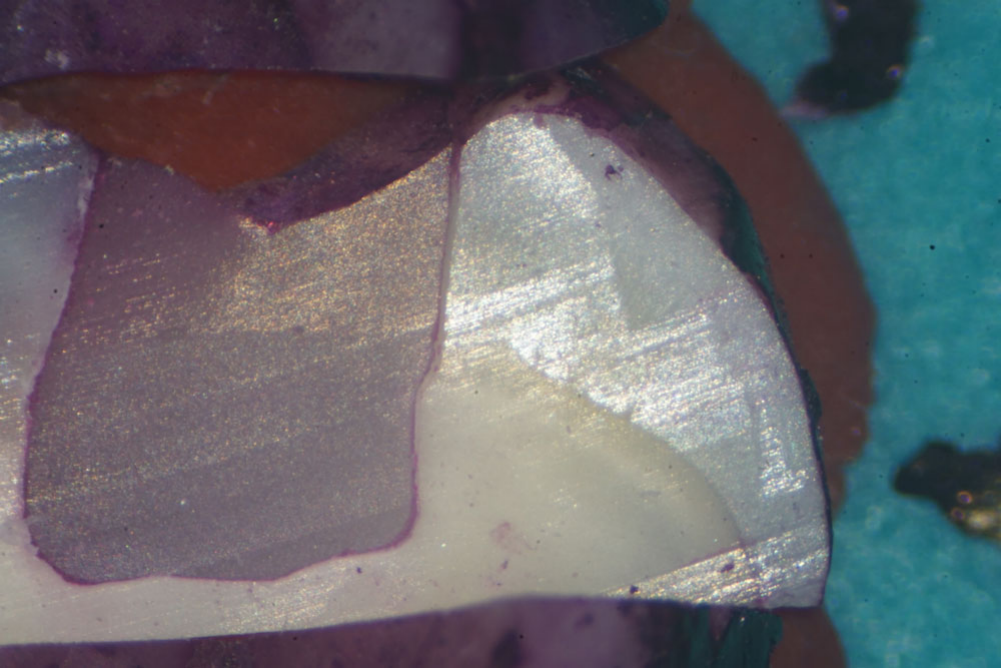
Showing an example of “score 3,” or maximum infiltration

Microleakage was evaluated for each group under an optical microscope at a magnification of ×20 and rated using a qualitative scale for evaluating the marginal sealing effectiveness of different restoration techniques. Each tooth section was rated by five independent observers, clinical practitioners with at least 5 years of experience each. The study aims and methods were presented to the observers and the scoring methodology was explained to them before the task. Observers were blind to what tooth section they were rating, so as to eliminate bias. After every 20 sections examined, a 10 ‐min break was granted to eliminate observer fatigue. For added clinical relevance of the study, observers also quantified whether infiltration reached just the enamel or it penetrated into dentin, using a second scoring method presented in Table 4. As an example of the scoring method, Figure 1 shows a specimen with no infiltration between the tooth walls and the restoration, Figure 2 shows slight infiltration in the enamel when looking at the lingual cusp (on the right of the image), that reaches only 1/3 or less of the cavity depth. In Figure 3, infiltration of the buccal cusp (on the left of the image) reaches ½ of the cavity, but still not touching the dentin. Pigmentation of the enamel can also be observed. Figure 4 shows deep infiltration, the dye circling the perimeter of the composite resin restoration. The cavity is infiltrated in its full depth.

**Table 4:** Additional scoring method, used for better clinical integration of the results

Score	Quality
0	*n* (no infiltration)
1	*e* (infiltration up to enamel/dentin junction)
2	*e*/*d* (infiltration up to or past the enamel dentin junction)
3	*d* (infiltration past the enamel/dentin junctions)

The data were analyzed using the one‐way analysis of variance test using SPSS software (IBM SPSS Statistics for Windows, Version 26.0: IBM Corp.)

## 3 RESULTS

The degree of dye infiltration between the restoration and the tooth wall was analyzed by five independent observers so as to eliminate any observer‐related shortcomings. To evaluate the internal consistency of the observers' score, an internal reliability test was run, with an Interclass reliability coefficient of 0.832, which showed very high agreement between the observers. Also, for a broader view of the marginal sealing of the samples, each tooth section was scored in four sites, which were averaged to a single score per tooth.

When comparing the five groups, no statistically significant difference could be found between the degree of infiltration and the restoration techniques used. 90.2% of the specimens were infiltrated with dye, of which 68.2% reached dentin.

The group that showed the lowest infiltration scores was Group A (Packable Bulk technique + simultaneous modeling) with a mean of 1.41 (±0.878). The group that showed the highest scores was Group E, corresponding to successive cusp build‐up with brush adaptation under magnification. Mean scores for all the compared groups are presented in Table 5.

**Table 5:** Visual representation of the statistics

Showing means + SD values and the statistical analysis of infiltration between different restoration techniques				
Group	*N*	Mean ± SD	ANOVA	
			*F*	Sig.
A	20	1.41 ± 0.878	.712	.586
B	20	1.66 ± 0.681		
C	20	1.46 ± 0.679		
D	20	1.75 ± 0.853		
E	20	1.645 ± 0.691		

*Note*: *p* < .05 considered as statistically significant. Higher mean scores indicate more microleakage.

Abbreviation: ANOVA, analysis of variance.

## 4 DISCUSSION

The null hypothesis of this paper proposed that the marginal integrity of posterior composite restorations, expressed through the degree of dye infiltration, would not be influenced by the technique in which the last occlusal layer of composite is placed. Our findings validate this hypothesis, since there was no statistical difference between layering techniques when evaluating the degree of dye infiltration between the restorative material and the cavity walls. These findings are of significant importance, showing that irrespective of the technique chosen to fill the cavity, some degree of infiltration is inevitable, although the bulk technique and the successive cusp build‐up show a slight improvement in sealing, as described by other papers (Orłowski et al., 2015; Zorzin et al., 2015).

Our findings regarding Group A echo with Sarfi et al. findings, where no statistically significant difference was found in microleakage between two incremental techniques (Sarfi et al., 2017) but are different from the findings of Tjan et al. where the bulk technique showed more leakage than the incremental techniques (Tjan et al., 1992). Our findings show that bulk composites have evolved over the years and can now provide similar marginal integrity to other types of composite materials.

In recent years, authors described the stamp technique for improving efficiency and patient compliance in regard to dental treatments (Perrin et al., 2013). A series of case reports proved this technique can reliably reconstitute occlusal cavities and Class 2 as well (Alshehadat et al., 2016; Araujo et al., 2009). Our results showed that this technique which was used in Group B could be sustainable and comparable by means of marginal infiltration with other standard techniques such as the successive cusp build‐up technique. By this point, no research paper focused on the stamp technique's marginal sealing efficiency, thus future research in this field is needed.

The successive cusp build‐up technique, representing Group C in our experiment can be regarded as a modified oblique incremental filling technique, for recreating the occlusal anatomy of molars with high precision (Step by step protocol, 2021). Although the oblique incremental technique was widely studied for its performance in reducing the C‐factor and improving the marginal sealing ability (Bugalia et al., 2011; Chandrasekhar et al., 2017; Tjan et al., 1992) no studies so far explored the successive cusp build‐up technique and marginal sealing. This technique provides a more didactic protocol for recreating the occlusal anatomy and it may even shorten the duration of the treatment because less time is spent with occlusal adaptation. In our study, this technique also showed the lowest scores after the bulk technique group, although being not statistically significant.

Composite brushes are generally used in day‐to‐day practice when modeling the occlusal anatomy and for better manipulation and adaptation of the composite resin. Brushes offer improved maneuverability of the composite and can be used to smoothen the texture of the material before polymerization (Layering composites just got easier, 2018). Through this experiment, our aim was to assess whether composite brushes could also improve marginal integrity and reduce microleakage. From our findings, no statistically significant results could be concluded, but the group which was restored using a modeling brush and magnification showed 99% of specimens infiltrated, although only up to the enamel/dentin junction. The group which was restored without magnification showed fewer infiltrated specimens (87%), but the infiltration was deep beyond the enamel/dentin junction. This may be due to the fact that, when working with the microscope, the magnified image of the operatory field leads to low amplitude movements of the operator. These movements might translate into low pressure when compacting the composite material and thus superficial gaps are more likely to appear. Consequently, better visibility aids in the better adaptation of the flowable bulk material on all walls of the cavity, which can explain the fact that when working under magnification, infiltration stops at the enamel/dentin junction.

When comparing Groups C and D, where the only variable was the use of brush adaptation, a 22% decrease in infiltration at the enamel/dentin junction could be observed when using a brush. Also, the stamp technique showed fewer specimens infiltrated up to the enamel/dentin junction. These might be due to the fact that the pressure of compaction created with the stamp and the brush led to a tight seal at the cavo‐superficial angles of the restoration.

The clinical relevance of this in vitro experiment can be drawn from the fact that 68.2% of the samples were visibly infiltrated as far as to touch dentin. The fuchsin dye contains compounds with larger molecules than the silver ion (Bonilla et al., 2012), which is smaller than a bacterium, therefore it could be reasoned that infiltration was even deeper. Once bacteria reach dentin, there is a higher chance of secondary caries forming in the future and the need for a new restoration.

These results are of high significance for clinicians because they indicate that no matter the technique used for restoring occlusal cavities, the outcome will be predictable and comparable between techniques. As limitations of our study, a larger sample size could be mentioned for leading to a more significant result. Also, the techniques used in this experiment were highly operator‐sensitive, but this risk was significantly reduced by using only one operator for the restorative procedure. Even having only one operator, the technique is sensitive due to human‐related inconsistencies.

As new techniques develop, future research is needed to support the argument that microleakage can be overcome.

## 5 CONCLUSION

Within the limitations of this study, we conclude that the layering techniques examined do not differ significantly by means of marginal infiltration and that the use of a modeling brush and magnification might improve marginal adaptation, while reducing microleakage, but not statistically significant.

## AUTHOR CONTRIBUTIONS

Bud M. Gheorghe conceived and designed the study and its aims, together with Pop R. Corneliu and Pricope Razvan. Pop R. Corneliu and Pricope Razvan carried out the experiment, helped by Voina Andrada. Voina Andrada and Mesaros Anca were in charge of providing the materials for the experiment and assuring their quality. Lucaciu Ondine, Delean Ada, and Cîmpean Sanda supervised the experiment and the findings of this work. All authors discussed the results and contributed to the final manuscript.

## CONFLICT OF INTEREST

The authors declare no conflict of interest.

## Data Availability

The data that support the findings of this study are available from the corresponding author upon reasonable request.

## References

[cre2664-bib-0001] Alshehadat, S. A. , Halim, M. S. , Carmen, K. , & Fung, C. S. (2016). The stamp technique for direct Class II composite restorations: A case series. Journal of Conservative Dentistry: JCD, 19(5), 490–493. 27656074 10.4103/0972-0707.190021 PMC5026115

[cre2664-bib-0002] Araujo, E. M. , De Goes, M. F. , & Chan, D. C. (2009). Utilization of occlusal index and layering technique in class I silorane‐based composite restorations. Operative Dentistry, 34(4), 491–496. 19678456 10.2341/08-004-T

[cre2664-bib-0003] Askar, H. , Krois, J. , Göstemeyer, G. , Bottenberg, P. , Zero, D. , Banerjee, A. , & Schwendicke, F. (2020). Secondary caries: What is it, and how it can be controlled, detected, and managed?”. Clinical Oral Investigations, 24(5), 1869–1876. 32300980 10.1007/s00784-020-03268-7

[cre2664-bib-0004] Bonilla, E. D. , Stevenson, R. G. , Caputo, A. A. , & White, S. N. (2012). Microleakage resistance of minimally invasive class I flowable composite restorations. Operative Dentistry, 37(3), 290–298. 22313270 10.2341/11-106-L

[cre2664-bib-0005] Bud, M. , Jitaru, S. , Lucaciu, O. , Korkut, B. , Dumitrascu‐Timis, L. , Ionescu, C. , Cimpean, S. , & Delean, A. (2021). The advantages of the dental operative microscope in restorative dentistry. Medicine and Pharmacy Reports, 94(1), 22–27. 33629044 10.15386/mpr-1662 PMC7880065

[cre2664-bib-0006] Bud, M. , Pricope, R. , Pop, R. C. , Onaca, R. , Swerts, P. J. , Lucaciu, O. , & Delean, A. (2021). Comparative analysis of preclinical dental students' working postures using dental loupes and dental operating microscope. European Journal of Dental Education, 25(3), 516–523. 33180967 10.1111/eje.12627

[cre2664-bib-0007] Bugalia, A. , Usha, G. , Karthik, J. , Rao, R. , & Vedhavathi, B. (2011). Effect of four different placement techniques on marginal microleakage in class II composite restorations: An in vitro study. World Journal of Dentistry, 2(2), 111–116.

[cre2664-bib-0008] Chandrasekhar, V. , Rudrapati, L. , Badami, V. , & Tummala, M. (2017). Incremental techniques in direct composite restoration. Journal of Conservative Dentistry: JCD, 20(6), 386–391. 29430088 10.4103/JCD.JCD_157_16 PMC5799982

[cre2664-bib-0009] Cumerlato, C. B. F. , Demarco, F. F. , Barros, A. J. D. , Peres, M. A. , Peres, K. G. , Morales Cascaes, A. , de Camargo, M. , da Silva dos Santos, I. , Matijasevich, A. , & Corrêa, M. B. (2019). Reasons for direct restoration failure from childhood to adolescence: A birth cohort study. Journal of Dentistry, 89, 103183. 31449840 10.1016/j.jdent.2019.103183

[cre2664-bib-0010] Demarco, F. F. , Collares, K. , Correa, M. B. , Cenci, M. S. , MORAES, R. , & OPDAM, N. J. (2017). Should my composite restorations last forever? Why are they failing?”. Brazilian Oral Research, 31(suppl 1), 92–99. 10.1590/1807-3107BOR-2017.vol31.0056 28902236

[cre2664-bib-0011] Geerts, S. , Bolette, A. , Seidel, L. , & Guéders, A. (2012). An in vitro evaluation of leakage of two etch and rinse and two self‐etch adhesives after thermocycling. International Journal of Dentistry, 2012, 852841. 22675358 10.1155/2012/852841 PMC3364560

[cre2664-bib-0012] Ghulman, M. A. (2011). Effect of cavity configuration (C factor) on the marginal adaptation of low‐shrinking composite: A comparative ex vivo study. International Journal of Dentistry, 2011, 159749. 21949664 10.1155/2011/159749 PMC3178442

[cre2664-bib-0013] Goldstein, G. R. (2010). The longevity of direct and indirect posterior restorations is uncertain and may be affected by a number of dentist‐, patient‐, and material‐related factors. The Journal of Evidence‐Based Dental Practice, 10(1), 30–31. 20230962 10.1016/j.jebdp.2009.11.015

[cre2664-bib-0014] Hayashi, J. , Tagami, J. , Chan, D. , & Sadr, A. (2020). New bulk‐fill composite system with high irradiance light polymerization: Integrity and degree of conversion. Dental Materials, 36(12), 1615–1623. 33168226 10.1016/j.dental.2020.10.012

[cre2664-bib-0015] Hirata, R. , Kabbach, W. , de Andrade, O. S. , Bonfante, E. A. , Giannini, M. , & Coelho, P. G. (2015). Bulk fill composites: An anatomic sculpting technique. Journal of Esthetic and Restorative Dentistry, 27(6), 335–343. 26177219 10.1111/jerd.12159

[cre2664-bib-0016] Layering composites just got easier . (2018). British Dental Journal , 224(7), 552.

[cre2664-bib-0017] Leknius, C. , & Geissberger, M. (1995). The effect of magnification on the performance of fixed prosthodontic procedures. Journal of the California Dental Association, 23(12), 66–70. 9052031

[cre2664-bib-0018] Liebenberg, W. H. (1996). Successive cusp build‐up: An improved placement technique for posterior direct resin restorations. Journal (Canadian Dental Association), 62(6), 501–507. 8752648

[cre2664-bib-0019] Orłowski, M. , Tarczydło, B. , & Chałas, R. (2015). Evaluation of marginal integrity of four bulk‐fill dental composite materials: In vitro study. Scientific World Journal, 2015, 2015. 10.1155/2015/701262 PMC4385685 25874254

[cre2664-bib-0020] Park, J. , Chang, J. , Ferracane, J. , & Lee, I. B. (2008). How should composite be layered to reduce shrinkage stress: Incremental or bulk filling?”. Dental Materials, 24(11), 1501–1505. 18433857 10.1016/j.dental.2008.03.013

[cre2664-bib-0021] Perrin, K. P. , Brigitte, Z. F. , Daniel, J. , Adrian, L. , Zimmerli, B. , Jacky, D. , Lussi, A. , Helbling, C. , & Ramseyer, S. (2013). Die stempeltechnik für direkte kompositversorgungen. Schweizer Monatsschrift für Zahnmedizin = Revue mensuelle suisse d'odonto‐stomatologie = Rivista mensile svizzera di odontologia e stomatologia/SSO, 123(2), 111–129. 23512289

[cre2664-bib-0022] Reddy, P. , Jain, V. , Kaushik, M. , Roshni , Mehra, N. , Rana, R. , & Yadav, M. (2017). Assessment of marginal integrity of proximal composite resin restorations performed with or without magnification. Journal of Clinical and Diagnostic Research, 11(12), ZC01–ZC04. 10.7860/JCDR/2017/29860/10921

[cre2664-bib-0023] Reher, V. , Reher, P. , Peres, K. G. , & Peres, M. A. (2021). Fall of amalgam restoration: A 10‐year analysis of an Australian university dental clinic. Australian Dental Journal, 66(1), 61–66. 33197295 10.1111/adj.12807

[cre2664-bib-0024] Sarfi, S. , Bali, D. , & Grewal, M. S. (2017). Effect of different layering techniques on microleakage of nanofilled composite in Class I restorations: An in vitro study. Journal of the International Clinical Dental Research Organization, 9(1), 8.

[cre2664-bib-0025] Scolavino, S. , Paolone, G. , Orsini, G. , Devoto, W. , & Putignano, A. (2016). The simultaneous modeling technique: Closing gaps in posteriors. The International Journal of Esthetic Dentistry, 11(1), 58–81. 26835524

[cre2664-bib-0026] Shah, P. K. , & Stansbury, J. W. (2021). Photopolymerization shrinkage‐stress reduction in polymer‐based dental restoratives by surface modification of fillers. Dental Materials, 37(4), 578–587. 33573842 10.1016/j.dental.2021.01.013 PMC7996127

[cre2664-bib-0027] Step by step protocol: Restoring the first upper molar . Retrieved September 26, 2021, from https://www.styleitaliano.org/restoring-the-first-upper-molar/

[cre2664-bib-0028] Tjan, A. H. , Bergh, B. H. , & Lidner, C. (1992). Effect of various incremental techniques on the marginal adaptation of class II composite resin restorations. The Journal of Prosthetic Dentistry, 67(1), 62–66. 1548611 10.1016/0022-3913(92)90051-b

[cre2664-bib-0029] Usha, H. L. , Kumari, A. , Mehta, D. , Kaiwar, A. , & Jain, N. (2011). Comparing microleakage and layering methods of silorane‐based resin composite in class V cavities using confocal microscopy: An in vitro study. Journal of Conservative Dentistry: JCD, 14(2), 164–168. 21814359 10.4103/0972-0707.82624 PMC3146110

[cre2664-bib-0030] Vanishree, H. S. , Shanthala, B. M. , & Bobby, W. (2015). The comparative evaluation of fracture resistance and microleakage in bonded amalgam, amalgam, and composite resins in primary molars. Indian Journal of Dental Research, 26(5), 446–450. 26672412 10.4103/0970-9290.172019

[cre2664-bib-0031] Veloso, S. , Lemos, C. , de Moraes, S. , do Egito Vasconcelos, B. C. , Pellizzer, E. P. , & de Melo Monteiro, G. Q. (2019). Clinical performance of bulk‐fill and conventional resin composite restorations in posterior teeth: A systematic review and meta‐analysis. Clinical Oral Investigations, 23(1), 221–233. 29594349 10.1007/s00784-018-2429-7

[cre2664-bib-0032] Zhou, X. , Huang, X. , Li, M. , Peng, X. , Wang, S. , Zhou, X. , & Cheng, L. (2019). Development and status of resin composite as dental restorative materials. Journal of Applied Polymer Science, 136(44), 48180.

[cre2664-bib-0033] Zorzin, J. , Maier, E. , Harre, S. , Fey, T. , Belli, R. , Lohbauer, U. , Petschelt, A. , & Taschner, M. (2015). Bulk‐fill resin composites: Polymerization properties and extended light curing. Dental Materials, 31(3), 293–301. 25582061 10.1016/j.dental.2014.12.010

